# Structural and Energetic Characterization of the Ankyrin Repeat Protein Family

**DOI:** 10.1371/journal.pcbi.1004659

**Published:** 2015-12-21

**Authors:** R. Gonzalo Parra, Rocío Espada, Nina Verstraete, Diego U. Ferreiro

**Affiliations:** Protein Physiology Lab, Dep de Química Biológica, Facultad de Ciencias Exactas y Naturales, UBA-CONICET-IQUIBICEN, Buenos Aires, Argentina; University College London, UNITED KINGDOM

## Abstract

Ankyrin repeat containing proteins are one of the most abundant solenoid folds. Usually implicated in specific protein-protein interactions, these proteins are readily amenable for design, with promising biotechnological and biomedical applications. Studying repeat protein families presents technical challenges due to the high sequence divergence among the repeating units. We developed and applied a systematic method to consistently identify and annotate the structural repetitions over the members of the complete Ankyrin Repeat Protein Family, with increased sensitivity over previous studies. We statistically characterized the number of repeats, the folding of the repeat-arrays, their structural variations, insertions and deletions. An energetic analysis of the local frustration patterns reveal the basic features underlying fold stability and its relation to the functional binding regions. We found a strong linear correlation between the conservation of the energetic features in the repeat arrays and their sequence variations, and discuss new insights into the organization and function of these ubiquitous proteins.

## Author Summary

Some natural proteins are formed with repetitions of similar amino acid stretches. Ankyrin-repeat proteins constitute one of the most abundant families of this class of proteins that serve as model systems to analyze how variations in sequences exert effects in structures and biological functions. We present an in-depth analysis of the ankyrin repeat protein family, characterizing the variations in the repeating arrays both at the structural and energetic level. We introduce a consistent annotation for the repeat characteristics and describe how the structural differences are related to the sequences by their underlying energetic signatures.

## Introduction

Ankyrin Repeat Proteins (ANKs) are usually described as composed of linear arrays of tandem copies of a ∼33 residues length motif with a canonical helix-loop-helix-*β*-hairpin/loop fold. Being one of the most common protein motifs in nature [1], these molecules are known to function as specific protein-protein interactors. The diversity of unrelated molecules with which they interact is reflected at the many cellular processes in which they are involved [1, 2]. The Notch receptor is a key molecule for metazoan development, involved in cell-cell signaling. Notch has been associated in many types of cancer and several Notch inhibitors are being evaluated for cancer treatments [3]. P16 is a tumor suppressor protein that regulates cell cycle, and mutations on P16 protein are related to several malignancies [4]. The I*κ*B family constitutes a group of related molecules that act as inhibitors of the NF-*κ*B transcription factors. The I*κ*B/NF-*κ*B system is involved in many cellular processes such as cell adhesion, immune and proinflammatory responses and apoptosis [5] as well as in Alzheimer’s disease, diabetes, AIDS, and many types of cancer [6].

Having an elongated architecture, interactions between residues are confined within repeats or between adjacent repeats. These molecules constitute useful models to study sequence-structure-function relationships. However, this non-globular fold represents new challenges [7]. Divergence in the primary structure can be high in comparison to the tertiary structure, with sequence identities between repeats lying on the twilight zone of sequence alignments [8], thus hindering the analysis of repeats at the family level.

We developed and applied a systematic method to consistently identify and annotate the structural repetitions over the members of the complete Ankyrin Repeat Protein Family. We consistently defined the repeats along all the members of the family, and performed comparative studies. We statistically characterized the number of repeats, the folding of the repeat-arrays, their structural variations, insertions and deletions. An energetic analysis of the local frustration patterns reveal the basic features underlying fold stability and its relation to the functional binding regions.

## Materials and Methods

### Data collection

All proteins with at least one detectable ANK repeat were retrieved from the Protein Data Bank (PDB, http://www.rcsb.org/ as it was at May 2014). Repeats were detected by using the hmmsearch module from HMMER (http://hmmer.wustl.edu) and the Hidden Markov Models (HMMs) from Pfam that correspond to the ANK Clan (Pfam ID = CL0465). This Clan is composed of 7 individual domains which are supposed to be evolutionary related (Ank, ID: PF00023; Ank2, ID:PF12796; Ank3, ID: PF13606; Ank4, ID: PF13637; Ank5, ID: PF13857; DUF3420, ID: PF11900; DUF3447, ID: PF11929). We merged the results for which at least one hit was found to any of the HMMs in the ANK Clan. To avoid missing ANKs which could have extremely divergent sequence to be detected by any HMM in the ANK Clan, we looked for structures using the TopSearch application [9]. TopSearch performs structural alignments between a given structure or fragment of it (query) and the entire PDB. We used three different queries: the protein 3ANK (PdbID: 1n0q,A), the protein 4ANK (PdbID: 1n0r,A), and the fragment belonging to the two internal repeats of 4ANK. No further structures were found with this strategy. At last, we obtained 164 PDB structures that contain 54 unique Uniprot entries (http://www.uniprot.org/) and 44 structures of designed ANKs proteins that do not have Uniprot entries associated (S1 Table). Among the 54 proteins, only 25 are related to a unique structure while the others are associated to 2–8 different PDB structures. We created a non redundant set of structures by selecting the one that maximized the coverage of its corresponding uniprot entry. 19 designed ANKs were selected according to different structural and sequence parameters, such as the number of repeats, construction method, number of variations with respect to the consensus.

Mapping between PDB structure chains and Uniprot entries was assigned from the PDBSWS resource [10] and their sequences were retrieved from the UniprotKB database.

#### TopMatch

We used the TopMatch tool [11] to perform structural alignments. Given a pair of protein structures, TopMatch generates an exhaustive list of partial alignments with the transformations (rotations and translations) that maximize the superposition of equivalent C^*α*^ atoms. The alignments are ranked according to the TopMatch score S, which provides a metric for structural similarity [12].

$$S=\sum_{i}^{L}e^{-r_{i}^{2}/\sigma^{2}}$$

#### Protein tiling and tileability

Based on TopMatch we have recently developed an algorithm that analyses how well a structure can be explained by its fragments [13]. Given a protein structure, we consider every continuous fragment of the polypeptide as a possible tile. By selecting a list containing the best alignments between each tile and the whole structure we define the associated *Tile Score*, Θ_*i*_, which represents the fraction of the structural space that can be covered by repetitions of the tile being considered:

$$\Theta_{i}=\frac{C_{i}-L_{ii}}{N-L_{ii}}$$ (1)

where *C*_*i*_ is the coverage given by the tile repetitions, *L*_*ii*_ is the length of the tile and *N* the total number of residues. Additionally, we have defined *Tileability*, *Ξ*, a score that measures the hierarchical organization of tiles at different lengths in the structure. We showed that proteins displaying some kind of symmetry (i.e rotational, translational or mixed) score higher than those with no evident repetitions or symmetric relationships among their subparts. *Ξ* is calculated as follows: for all tiles that are defined for a specific tile length *L*_*i*_, we take the maximum tile score Θ_*i*_ and then take the average over all *L*.

### Energetic local frustration

The energy landscapes of natural proteins are funnelled towards the native ensemble, in accordance with the Principle of minimal frustration [14]. However, energetic conflicts can remain in the native state, and these may have functional consequences for the dynamics and activity of the protein [40]. The frustration index, *Fi*, [15] allows to localize and quantify the energetics frustration present in protein structures. Given a pair of contacting residues in the native state, their interaction energy is compared to the energies that would be found by placing different residues in the same native location (mutational frustration index) or by creating a different environment for the interacting pair (configurational frustration index). When comparing the native energy to the energy distribution resulting from these decoys, the native contacts are classified as highly, neutrally or minimally frustrated according to how distant the native energy is with respect to the mean value of the decoys, taking into account the standard deviation from the distribution. An analogous approach used to calculate *Fi* for contacts can be used for residues. In this case, the set of decoys is constructed by shuffling the identity of only one residue, keeping all other parameters and neighboring residues in the native location, and evaluating the total energy change upon mutation, i.e integrating the interactions that the residue establishes with all its neighbors (single-residue-level frustration index). The algorithm is available at www.frustratometer.tk [16].

### Information content calculation

Information content obtained from multiple sequence alignments (MSA) measures the conservation of amino acids at specific positions. Also, the information content can be calculated according to the local frustration states (from the frustratometer algorithm on the structural level [16]). The Schneider’s approach [17] was used to compute the information content for both sequences and local frustration states.

The information content per position or per contact (R) can be thought as the reduction of uncertainty regards to the maximum possible (*H*_*max*_). The uncertainty observed in a system as defined by Shannon [18] is $H_{observed}=-\sum_{i=1}^{M}P_{i}log_{2}P_{i}$, where *P*_*i*_ is the probability that the system is in state i. In this work the states are the amino acids at characteristic positions (for sequences) and the frustration class of contacts (for local frustration). The probabilities are normalized, i.e $\sum_{i=1}^{M}P_{i}=1$, where M is the size of the alphabet (20 for amino acids, 3 for the frustration index). Therefore, the information content can be calculated as *R* = *H*_*max*_ − *H*_*observed*_.

Generally, it is considered that *H*_*max*_ is reached for a uniform distribution of states: then $P_{i}^{max}=1/M$ and *H*_*max*_ = *log*_2_(*M*). Nevertheless, if states are not equally likely to occur the probability distribution of states should be used to estimate *H*_*max*_. We set *H*_*max*_ = *log*_2_(20) for sequence IC calculations, and used the distribution of states reported by Ferreiro et al [15] to calculate the *H*_*max*_ for frustration IC calculations.

### Specific ANK Hidden Markov Models

The MSAs derived from structural alignments produced by the tiling procedure were used to generate HMMs (using the hmmbuild module from the HMMER suit) specific for each type of repeat, i.e N-terminal, internal or C-terminal. These HMMs are available in https://github.com/proteinphysiologylab/StructuralAnks_Parra2015. The hmmsearch module from HMMER can be used to look for hits to any of these models over specific sequences of interest.

## Results

### Overall view of the structural dataset

We collected and curated all ANKs structures from the PDB Data Bank, with a grand total number of 169 entries. Since many entries correspond to the same protein, we defined a non redundant dataset of 74 proteins, where 55 correspond to natural ANKs and 19 correspond to human-designed ANKs (see Methods). The structures are composed of 3 to 12 ANK repeats (S1 Table) with an overall *α*-solenoid fold architecture according to the classification defined by Kajava [19]. Although the majority of the structures are mainly composed of ANK arrays, 5 entries contain non ANK domains in the same chain (Murine Arf-GAP, PdbID: 1dcq,A; Human Arf-GAP, PdbID: 3jue,B; AnkX protein from *Legionella pneumophila*, PdbID: 4bet,A; Human Osteoclast-stimulating factor 1, PdbID: 3ehq,A and the Serine/threonine-protein phosphatase PP1-beta catalytic subunit from *Gallus gallus*, PdbID: 1s70,B).

**Fig 1:**
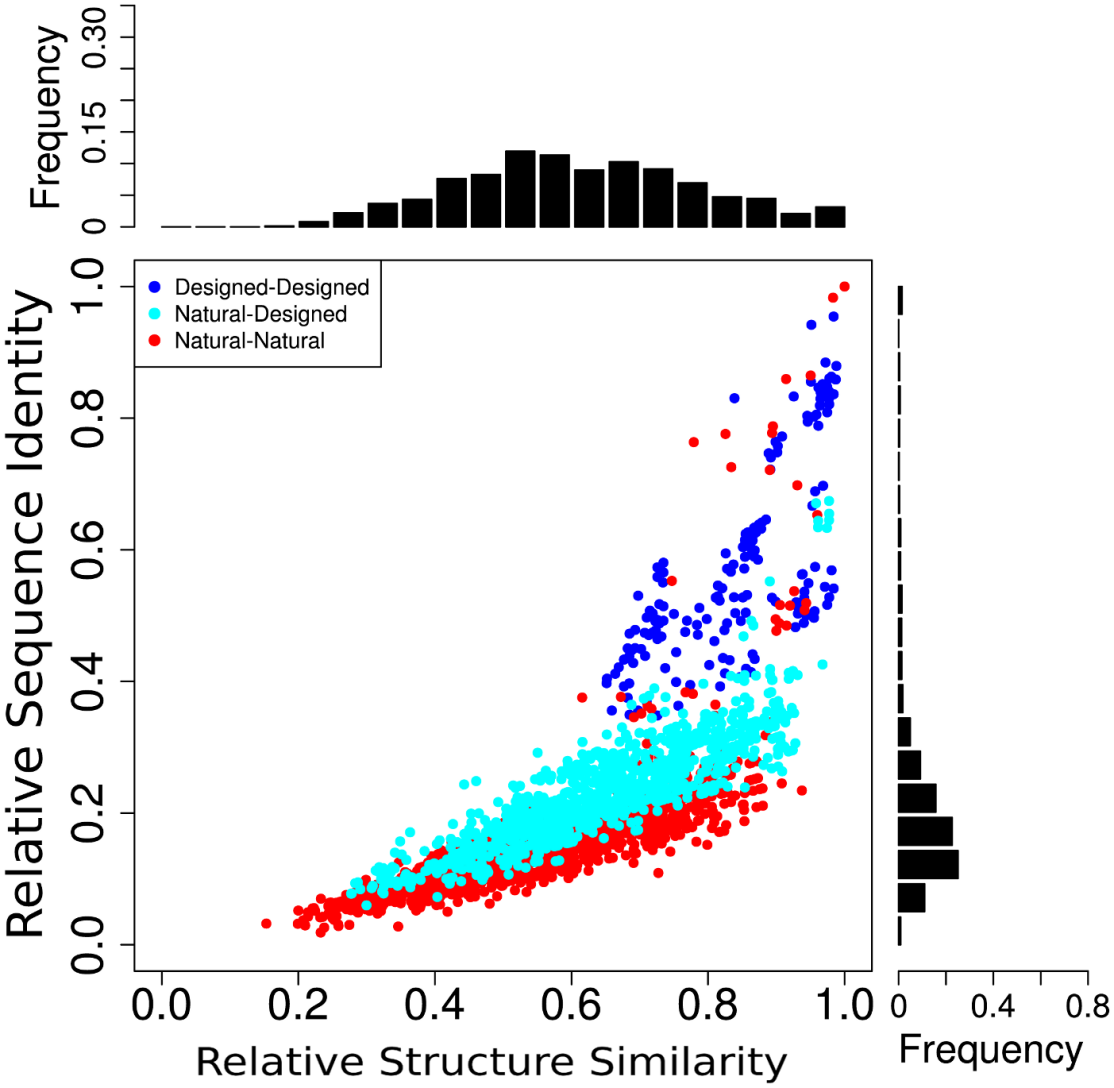
Structural comparison of Ankyrin proteins. Proteins from the non redundant dataset of Ankyrin proteins were structurally pairwise-aligned using TopMatch. The sequence identity (*relI*) and structural similarity (*relS*) parameters are compared. The distributions for each variable are shown in the upper and right side respectively.

ANKs appear to have very similar structures when observed globally, but subtle differences can be detected when looking carefully. We used the TopMatch tool [11] in order to analyze how (dis)similar the structures are, and pairwise aligned all the proteins from the non redundant dataset. TopMatch implements a metric for structural similarity [12], *S*, roughly equivalent to the number of residues that can be structurally aligned. A sequence identity value, *I*, is also calculated as the number of structurally aligned residues that are identical. Normalized values are used to correct *S* and *I* due to the differences in length of the aligned structures, with *la* and *lb* being the lengths of the two molecules being aligned, *relS* = 2 ⋅ *Sab*/(*la* + *lb*), as defined in [12], and *relI* = 2 ⋅ *Sab* ⋅ (*I*/100)/(*la* + *lb*). As a general trend, we observe that *relI* values are much lower (mean of 0.22 and sd = 0.16) than the corresponding *relS* values (mean of 0.7 and sd = 0.17), for two aligned proteins (Fig 1). Alignments between designed proteins are the ones that have the highest *relI* and *relS* values with mean values of 0.6 (sd = 0.17) and 0.85 (sd = 0.1) respectively. Alignments between natural proteins have a wider range of structural variability, with *relS* distribution centered at 0.55 (sd = 0.16) while *relI* is lower, with a distribution centered at about 0.17 (sd = 0.14). We found that there is a roughly linear correlation between sequence identity (*relI*) and structural variability (*relS*) distributions when aligning natural ANKs. The pairwise alignments show that despite a considerable sequence variability, structures with a *relI* as low as 0.2 can have a *relS* value of 0.8. In order to analyze which features of the family are captured by *relI* and *relS* a hierarchical analysis based on each of these measures was applied (S1 Fig).

A clustering analysis based on *relI* distinguishes the proteins mainly by their orthology and paralogy. Designed proteins segregate in a clearly separated cluster evidencing that natural proteins are mainly composed of non-consensus repeats. When the clustering analysis is performed using the *relS* values the clusters are mainly composed of proteins with similar number of repeats. We observe an outer group nucleating non-eukaryotic proteins, displaying long non-repetitive regions or belonging to a specific class of ion channels (TRPV family) which have strong structural modifications on their repeats. In order to find the repeat motif that better describes the family, we analyzed the dendrogram obtained from the clustering based on the *relS* parameter. Starting from the base, the leaves were collapsed in pairs from the bottom to the root. Each time a branch consisting of two members was collapsed, the common structure between the two is annotated. Once the whole dendogram is collapsed, the substructure that is common to all the dataset is obtained. The resulting regions match with the two internal repeats of the 4ANK protein [20].

### Repeat arrays are differentially organized

**Fig 2:**
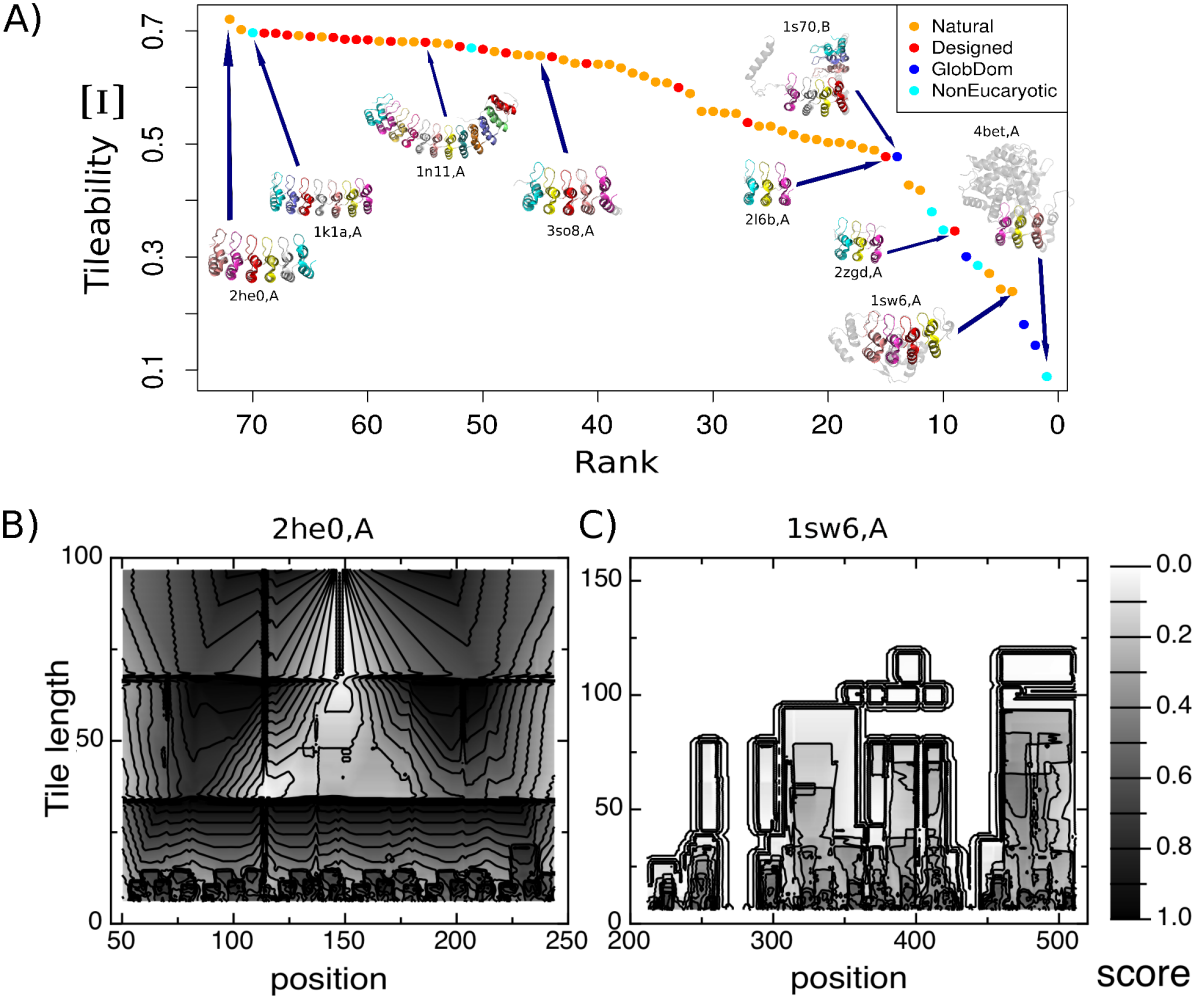
Symmetry of ANK domains. (A) Tileability values for the ANKs in the dataset. Higher values correspond to more regular structures. Lower values correspond to proteins containing structural perturbations that break the propagation of symmetry at higher length-scales by modifying the spatial arrangement of the repeating units. (B) Per residue tileability for Notch1 (PdbID: 2he0,A), the most symmetric protein in the dataset. (C) Per residue tileability for SWI6 (PdbID: 1sw6,A), one of the least symmetric proteins, due to a high occurrence of structural modifications in the repeats and insertions between them. The x-axis marks the residues in the structure, the y-axis represents the length of the fragments being considered in each case to tesselate the structures. The gray-scale represents the fraction of times in which a residue is part of a fragment that tessellates the protein.

Repeat proteins are the result of a collection of analogous motifs, organized in tandem arrays and related by different types of transformations between neighboring units (i.e. rotational, translational or screw [21, 22]). We have shown that a tileability score [13] captures how collectively periodic a structure is, taking into account how repetitive it is at different length-scales. Tileability scores for the ANKs in the non redundant dataset are shown in Fig 2A. Despite the fact that all these proteins share a common architecture with highly similar motifs disposed in tandem, small perturbations within or between repeats propagate to the global structure decreasing the overall symmetry. Proteins where the geometrical transformations between adjacent repeats are homogeneous along the array and do not present big insertions or deletions have high Tileabilities (e.g. Notch1, PdbID: 2he0,A; Ankyrin-1, PdbID: 1n11,A; ANKRA2, PdbID: 3so8,A). On the other side of the Tileability spectra we find proteins that hold a considerable proportion of non-repetitive regions (e.g. Serine/threonine-protein phosphatase PP1-beta catalytic subunit, PdbID: 1s70,B; AnkX, PdbID: 4bet,A) or display several structural perturbations (Cell cycle regulatory protein SWI6, PdbID: 1sw6,A).

Repeat proteins usually need modified versions of their terminal repeats in order to be soluble enough and to maintain their overall stability [23]. We separated the structurally detected repeats into three groups, i.e N-terminal, Internal and C-terminal repeats, and analyzed their structural and sequence properties. The first helix is usually extended in the case of the N-terminal repeats while in the case of C-terminal repeats, the second helix is extended (S2 Fig). In order to analyze how repetitive is a structure at different regions, we defined a per-residue function that measures how frequently a residue is covered by copies of fragments defined at all possible lengths and phases. Thus, we were able to locate which regions break the overall symmetry of the molecule. While some ANK structures, as the one corresponding to Notch1, are highly periodic for all tile sizes and at many phases (Fig 2B), some others are barely periodic with many regions that are non-repetitive as for SWI6 (Fig 2C). In this last example, we detected a region at the N-termini that has a combination of secondary structure elements that can be aligned with ANK repeats. This type of secondary structure arrangements, present also in other cases, may serve as non repetitive caps.

### Consistent definition of the Ankyrin repeat

**Fig 3:**
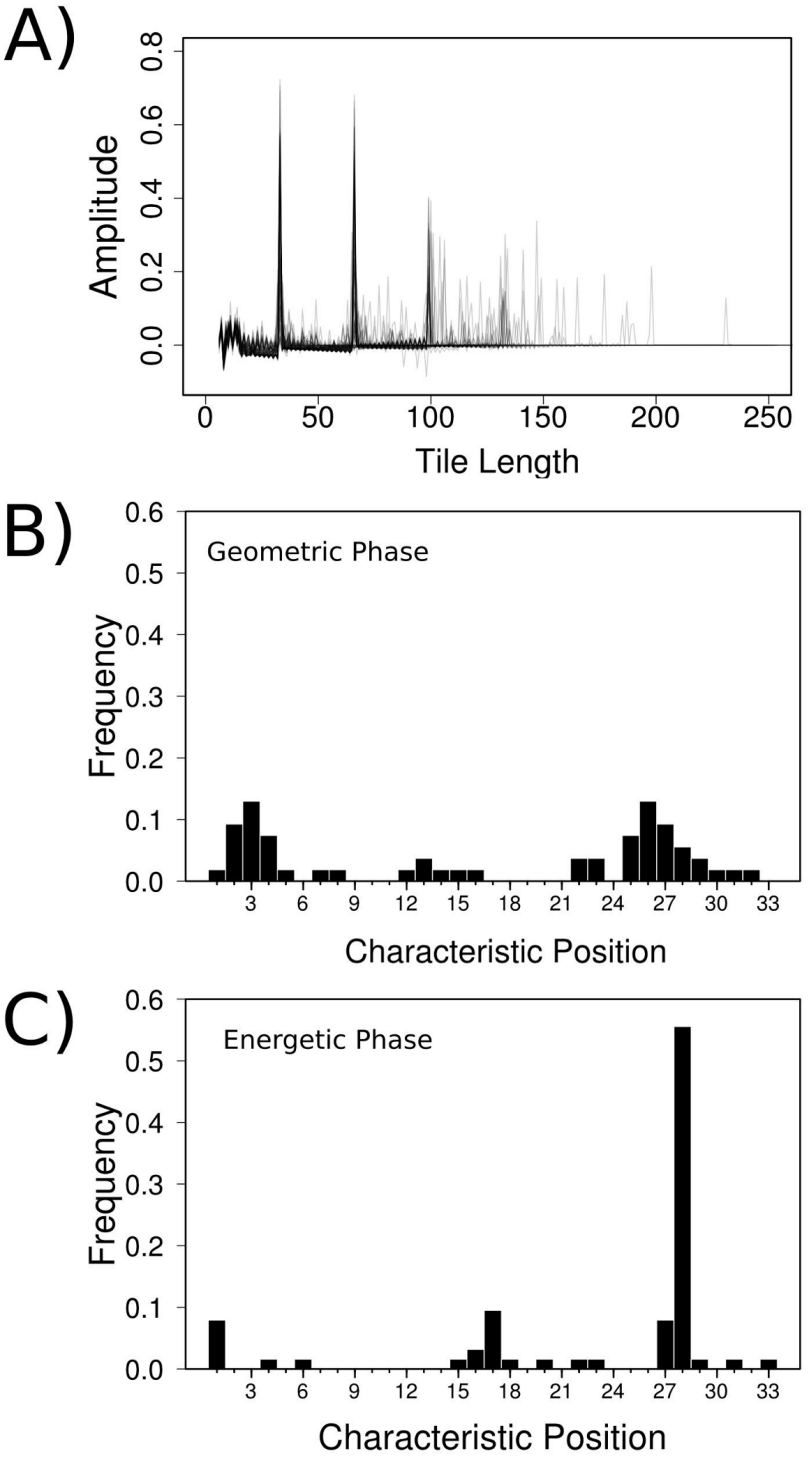
Defining the ANK repeat phase. (A) Frequency of structural repetitions as characterized by the *δ*-functions for ANK proteins. Most of the ANKs have their highest peak at a tile length of 33 residues. (B) Distribution of typical phases for the highest scoring fragments according to their tiling scores. The phase is shown relative to the ANK HMM in Pfam. These phases are defined by applying the tiling algorithm over the set of ANK structures, thus it is related to the geometry of the molecules. (C) Distribution of phases for the highest scoring fragments according to their relative foldability values. The phase is shown relative to the ANK HMM in Pfam. These phases are defined by calculating the foldability of the different tiles of the structure, thus it is related to the energetics of the different fragments.

Ankyrin domains are composed of a variable number of repeats that are arranged in tandem. In order to consistently define the structural repetitions we applied a structural search method that analyses the periodicities and repetitions in the protein structures [13]. Given their repetitive nature, ANK structures can be decomposed into minimal units whose copies can be translated and rotated to reconstruct the overall protein structure. We observe that most ANKs are markedly periodic, identifying fragments of 33 residues length as the peak of highest amplitude, and multiples of it (Fig 3A). This is in agreement with the repeat length reported by other studies [1, 24]. While a periodic pattern is evident, definition of limits between adjacent repeats (or periods) is not straight-forward.

To consistently annotate the repeats we considered the phase definition that better explains the structural observations in all the known ANKs. For each protein we selected the fragment of 33 residues length that has the highest coverage score and defined its phase by comparing it to the prototypical ANK Hidden Markov Model (HMM) deposited in the Pfam Database. We observed that in 70% of the structures, a multiplicity of fragments defined at different phases share the highest score. For the remaining 30% of the dataset a unique phase has the highest score, and there is a distribution of phases that are selected (Fig 3B). Thus there is not a clearly conserved geometric phase in natural ANK proteins.

Protein and repeat array stabilities may be related to the selection of a preferred phase of the repeat-unit. Each repeating unit has similar structural elements that interact with the nearest neighbors, and their folding process could be depicted by many folding funnels that merge upon interaction [25]. Following this idea, we took all the possible fragments of 33 residues length and calculated their relative foldability score, $\Theta =\Delta E/(\delta E\sqrt{N})$ [26]. We calculated the energy that corresponds to the sum of the internal interactions as measured by the AM/W energy function [27], and compared it to the mean energy of a set of N structures (ΔE) and its variance (*δ*E), for all the other fragments of 33 residues length in the ANK array. We took the fragment of 33 residues length with the highest Θ value in each protein, and assigned it a phase according to the ANK HMM. We observe that there is a highly conserved phase, starting at position 28 of the ANK HMM, for most of natural ANKs. Cases that deviate from this prevalent phase correspond mainly to designed proteins and members of the TRPV channels that constitute a specific subgroup of proteins where several structural modifications are present at individual repeats. Interestingly, proteins designed by Peng’s group in which covariations among the positions were respected [20], show a phase that agrees with most natural proteins (Fig 3C).

### Annotating and characterizing the repeats

**Fig 4:**
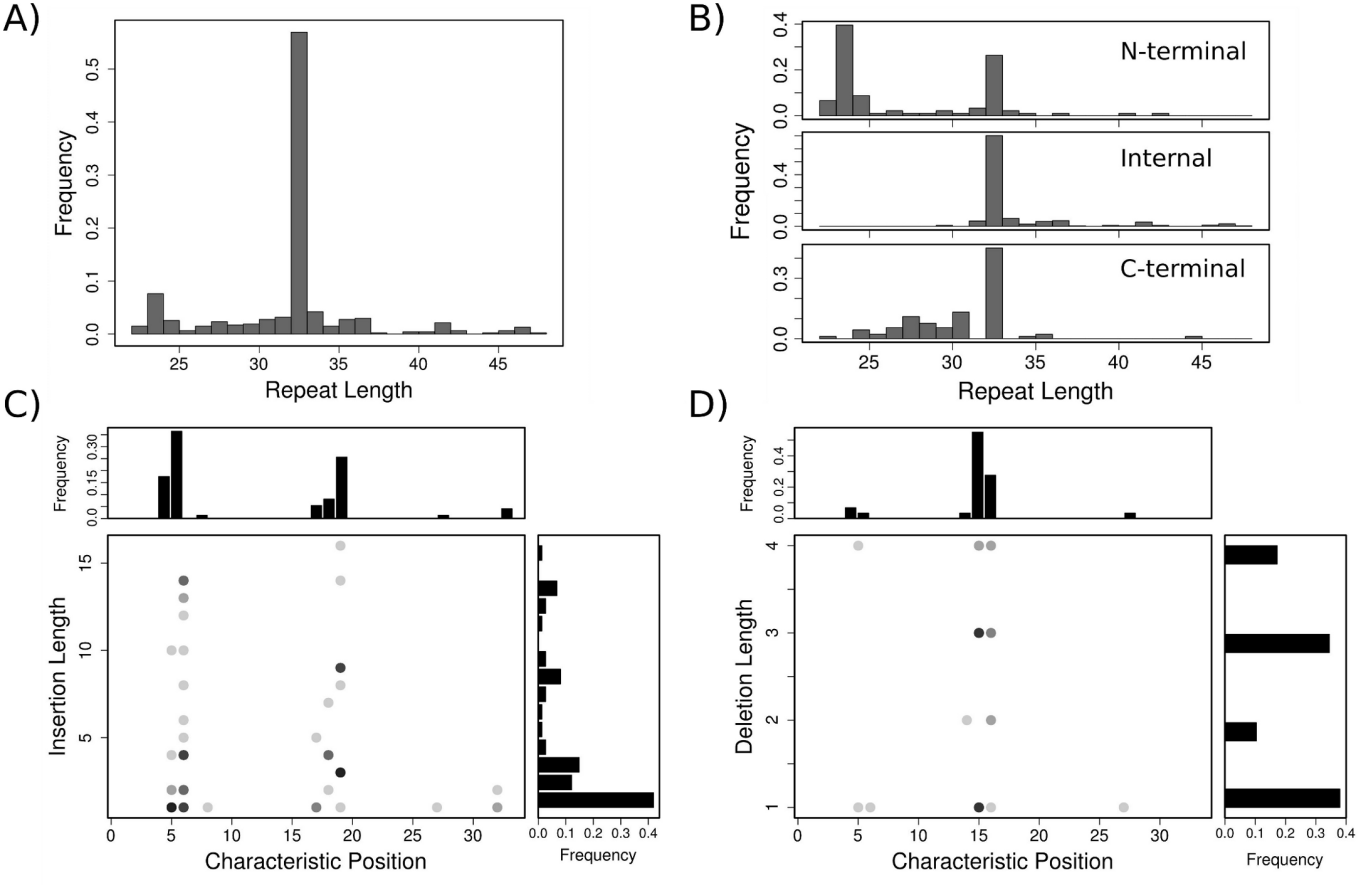
Overview of ANK domains structure. (A) Distribution of lengths for the repeats detected in structures. (B) Distribution of lengths for the N-terminal, internal and C-terminal repeats. (C) Distribution of deletions along the canonical positions in ANKs relative to the phase of the structural based HMM and their length. The grayscale for the dots is indicative of many points being superimposed. (D) Distribution of insertions along the canonical positions in the ANK repeat relative to the phase of the structural based HMM and their length.

Once both the period and the phase were unequivocally defined, we proceeded to detect and annotate the repeats on the ANKs comprised in the non redundant dataset (S1 Table). We selected the first internal repeat from the 4ANK protein, the one that maximizes the structural similarity, defined at the phase identified previously. We used this fragment to cover all other target proteins with repetitions of it. As a result, a set of structure-based sequence alignments between the 4ANK fragment and the matched subfragments within each target protein were obtained. For each alignment we redistributed the gaps satisfying that i) any gap modification for the query sequence was also applied to its target sequence and ii) globally align all the query sequences, which only differ in the number of gaps and location of them. Thus, all the target sequences became transitively aligned, producing a MSA using only structural information. Additionally, the pairwise analysis retrieved from the procedure allows to assign canonical positions to the repeating units as well as insertions and deletions, defined by those regions with gaps in either the query or target sequences, respectively. The length of the detected repeats range from 24 up to 48 residues (Fig 4A). Repeats that are located at the termini display largest deviations towards shorter lengths (Fig 4B). The most affected ones are the N-terminal, that lack the first *β*-hairpin region. In the case of the C-terminal repeats, deletions correspond in many cases to a shorter second helix. Insertions and deletions are not homogeneously distributed along the repeats but localized at specific regions. Insertions are mainly located at the *β*-hairpins and between the two *α*-helices (Fig 4C) and deletions are particularly located at the end of the first helix (Fig 4D). Both insertions and deletions show variable lengths, and they are not correlated with their locations. (Fig 4C and 4D). A total of 390 repeats were obtained with our structural method which is an increment of ∼18% respect with the 321 repeats that are detected using the ANK HMM in Pfam. If we consider only natural proteins, 311 repeats are detected in structure and 253 over sequences with the ANK HMM in Pfam. HMMs specific for the N-terminal, internal and C-terminal repeats were created from the specific MSAs for each type, using the hmmbuild module from the HMMER suit. 310 repeats are detected over the natural ANK sequences analyzed before, by just using the HMM derived from the internal repeats MSA.

### Energetic description of ANKs

ANKs are described as being specific protein-protein interactors [1]. Protein evolution is the result of different forces that select for their ability to fold into a stable structure and perform a biological function. In this sense, energetic conflicts present in the ANK repeat fold could be related with the ability of these proteins to efficiently bind their protein targets. We used the Frustratometer tool [16] in order to locate in the structures these energetic conflicts and quantify them by analyzing the different frustration indices [15].

#### Conservation of sequence and local frustration along the canonical structure of ankyrin repeats

**Fig 5:**
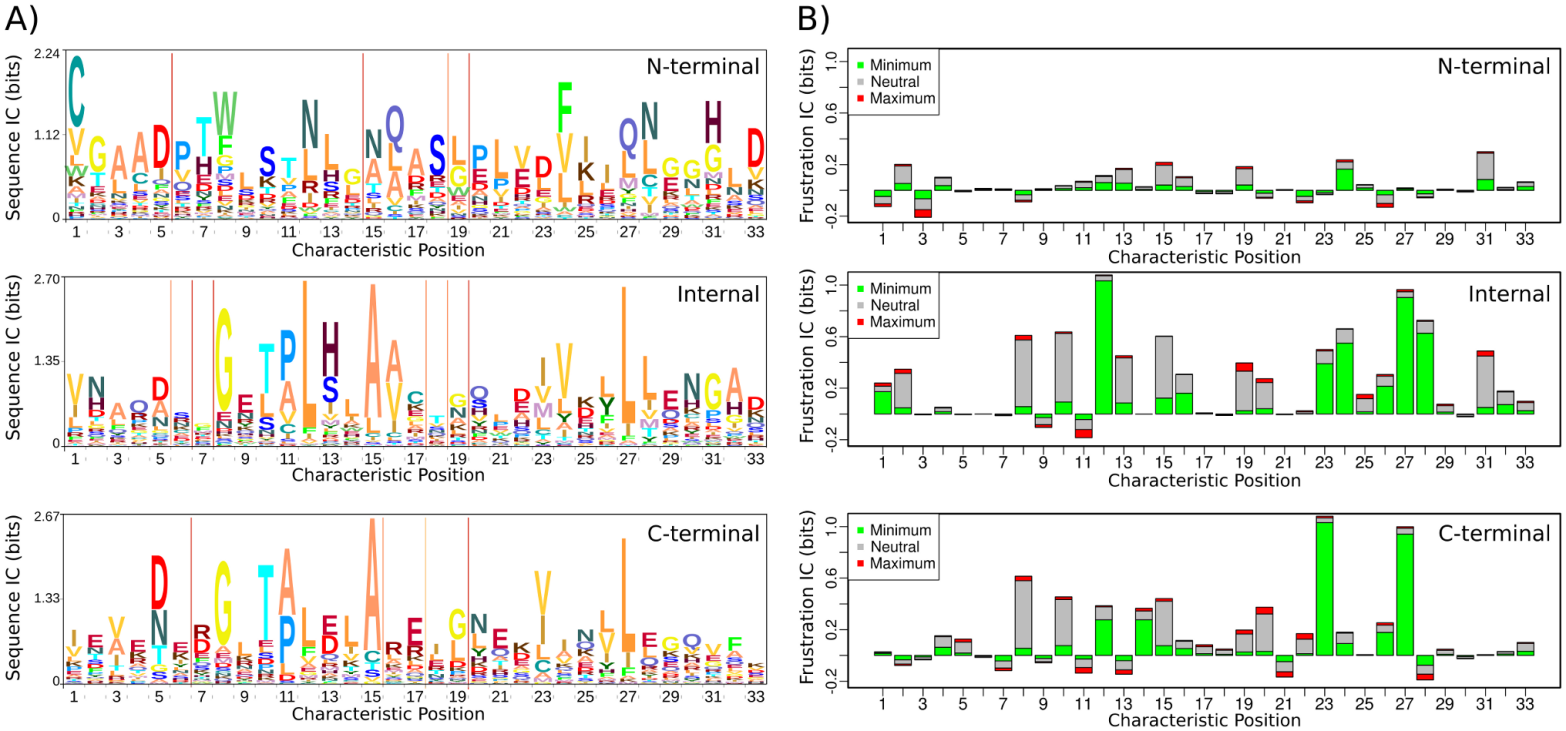
Conservation of local frustration in the Ankyrin Repeat Domains. A) Sequence logo corresponding to the hidden Markov models derived from indirect structural alignments for N-terminal repeats, Internal repeats and C-terminal repeats. B) Information Content for the conservation of frustration states according to the single residue level frustration index for N-terminal repeats, Internal repeats and C-terminal repeats. The total height of the stack represents the overall information content value of it. Contributions to the overall value of each frustration state at each position is represented by the proportion of each color to the stack.

We have shown that there are differences in the composition of secondary structure elements among the different types of repeats. We separated the repeats into the N-terminal, Internal and C-terminal groups and applied on them the same procedure as before in order to calculate an MSA, using only structural information. We calculated the corresponding HMMs for each repeat type and the corresponding sequence logos. There are marked differences among the sequence signatures for each type of repeat (Fig 5A). The internal repeat is the one with the most similar signature in respect to the ANK Pfam logo and the only one where the characteristic GXTPLHLA motif is highly conserved. On the contrary, this short motif seems to be completely absent in N-terminal repeats and semi conserved in the C-terminal repeats. Other positions highly conserved in the ANK Pfam Logo, as the Glycine at position 8, the Alanines at positions 15 and 16 and the Leucines at positions 27 and 28 are highly conserved in internal repeats, semi conserved in C-terminal repeats and not much conserved in the N-terminal ones. The sequence information content (sequence IC) for the entire profile, is the highest in the internal repeats profile (or conversely, the entropy is the lowest). The N-terminal repeats display the lowest sequence IC value while the C-terminal ones hold an intermediate value between the Internal and the N-terminal repeats. The differential exposure to solvent for these three types of repeats may explain some of this divergence [28] given the differences in their environments according to their position in the repetitive array.

The single-residue frustration index [15] distribution was calculated for each canonical position in the ANK repeats. Three classes of frustration states can be defined using this index, i.e minimally frustrated, neutral or highly frustrated. With the three classes of frustration an information content value (Frustration IC) was calculated for each canonical position for each repeat type (Fig 5B). Like the sequence IC, the frustration IC is the highest for the internal repeats and lowest for the N-terminal ones with differential profiles for the three repeat types. Although the overall frustration IC is higher for the internal repeats it is worth noting that positions 23 and 27, corresponding to a Valine and a Leucine respectively as the most conserved residues, show higher frustration IC values and may be related to structural requirements of C-terminal repeats. There are no positions, in any group, where the frustration IC is high and the highly frustrated state is the most informative one, meaning that there are not systematically frustrated positions along the ANK fold. Highly frustrated residues seem to be specific for the protein being analysed and may be directly related to its function. Positions that are allowed to be mutated, at internal repeats in DARPINs [29] which are specific protein-protein interactors, have low frustration IC values (positions 3,5,6,9,17,18 and 30 in the internal repeats profile in Fig 5B). However, there are other positions with low frustration IC values comparable to those. Specifically, there are 5 positions: position 4 which at Peng’s consensus sequence corresponds to a K, located at the *β*-hairpin, position 14 corresponding to the second Leucine within the prototypical GXTPLHLA motif, and positions 21, 22 and 29 that are located in the solvent exposed side from helix 2.

**Fig 6:**
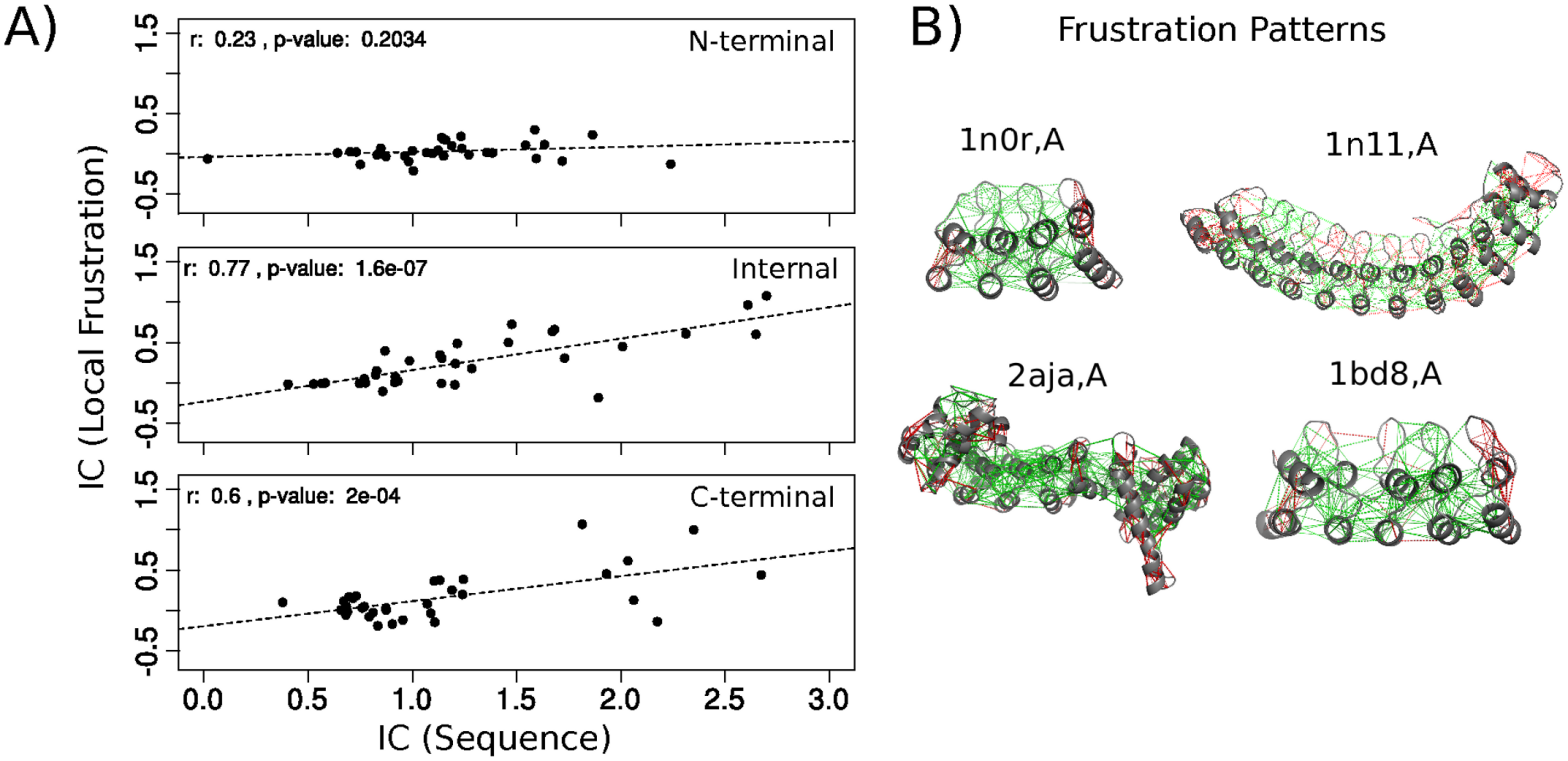
Local frustration and sequence conservation. A) Relation between information content per characteristic position in the ankyrin repeat derived from the conservation of the sequence and the conservation of the frustration state. There exists a positive correlation between the two variables with significant p-values (<0.05) for the Internal and C-terminal repeats. The correlation is not significant in the case of N-terminal repeats. B) Configurational frustration index plotted over representative structures of the ANKs family (the consensus protein 4ANK, PdbID: 1n0r,A; Ankyrin1, PdbID: 1n11,A; LegA1 protein from *Legionella pneumophila*, PdbID: 2aja,A and the P19INK4D CDK4/6 inhibitor, PdbID: 1bd8,A). The green lines correspond to interactions that are minimally frustrated, red lines correspond to highly frustrated interactions.

There is a positive and significant correlation between the sequence and frustration IC values for the internal and the C-terminal repeats (Fig 6A), and no significant correlation for the N-terminal case. This suggests that the more conserved an amino acid is at a defined position in the ANKs MSA, the greater the energy gap between the folded and unfolded states for the repeat. This establishes a direct connection between sequence conservation and structural stability. The lower frustration IC values for the terminal repeats, compared to the internal ones, suggests that these are less stable. This is in agreement with experiments showing that in many ANK proteins, the terminal regions are less stable and ‘fray’. It has been shown experimentally and computationally that for the tumor suppressor P16, the N-terminal repeat is the first to unfold [30, 31] which is also the case for the D34 protein [32]. The Notch protein has an N-terminal repeat that is partly disordered [33]. Gankyrin starts to fold at the C-terminal when isolated and changes its unfolding mechanism when in complex, where the N-terminal region is the first to unfold [34]. Structural flexibility at the terminal repeats of I*κ*B*α* is crucial for function. From the 6 repeats that conform the array, repeats 1, 5 and 6 are conformationally flexible [35]. It has been shown that the unfolded/folded transitions in repeat 6 are crucial to regulate the proteasomal degradation pathway of I*κ*B*α* [36]. Repeat 1 remains highly solvent accessible even after the complex is formed which would be critical to activate NF-*κ* by release of I*κ*B*α*. Furthermore, the conformational plasticity in repeats 5 and 6 along with the C-terminal PEST sequence is important in the stripping process of NF-*κ*B to dissociate it from the DNA in the nucleus and inactivate its transcriptional activity [37]. Thus, it is common to observe that terminal repeats are less stable than internal ones. This has a geometric origin, as terminal repeats have only one interface with the adjacent repeats, and formation of interfaces have been shown to be the most stabilizing factor. In addition, we observe that the terminal repeats are enriched in highly frustrated interactions that may reflect the fact these repeats are often involved in a biological activity.

#### Conservation of local frustration along the canonical contact maps of ankyrin repeats

**Fig 7:**
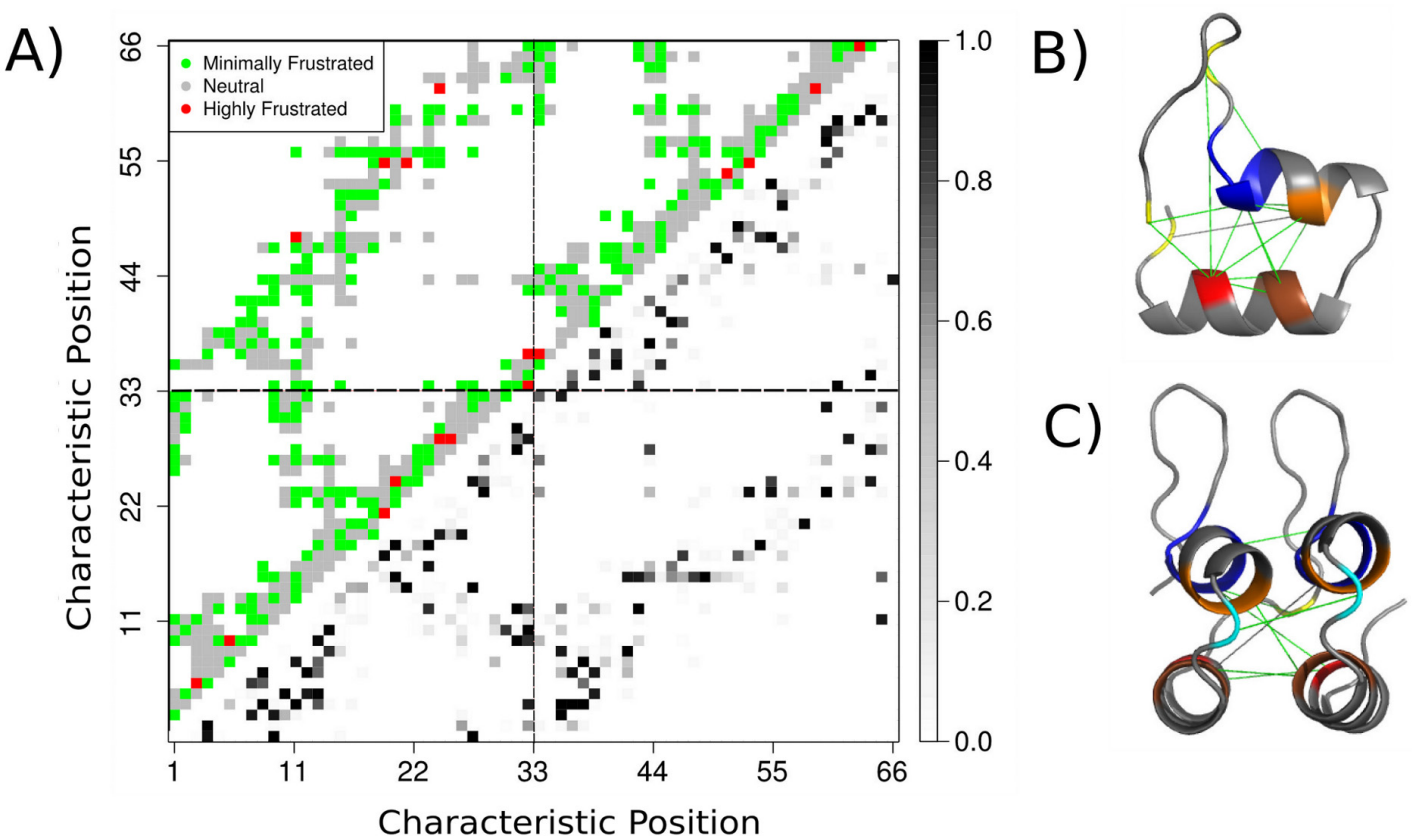
Conservation of local frustration at the contact-level. A) Contact map for a consecutive pair of ANK repeats. In the upper matrix the most informative state from the frustration IC calculations is shown. Red represents that the most informative state is the highly frustrated, gray is for the neutral state and green when the minimally frustrated state is the most informative one. Those values are calculated over the absolute counts that occur for each canonical interaction in the dataset. In the lower matrix the relative frequency of having a contact between two canonical positions in the repeats pair is shown in grey-scale where darker points mean that the interaction is present in a bigger fraction of the repeats being analyzed. B) Conservation of interactions in and between repeats: Contacts within the ankyrin repeat with a IC value higher than the highest interaction where the neutral state is the most informative. Residues involved in these conserved interactions are marked: Most conserved amino acid(s): Yellow: Position 8; G. Blue: Positions 11–14;TPLH. Orange: Positions 15–16; AA. Red: Positions 23–24; IV. C) Conservation of most informative contacts at the interface between repeats.

We calculated the Frustration IC for ANK repeat at the contact level. Examples of frustration maps on ANK structures are shown on Fig 6B. We took all pairs of internal repeats in order to analyze the interfaces between neighboring repeats. The relative frequencies of having or not a contact between two canonical positions in the ANKs pair contact map was calculated (Fig 7A, lower triangular matrix). The Frustration IC was evaluated for each canonical contact (see methods) and the more informative state was recorded (Fig 7A, upper triangular matrix). We weighted the Frustration IC value by the relative frequency of having that canonical contact in a pair of repeats from the dataset, and calculated the distributions according to which frustration state is the most informative one at each canonical contact (S3 Fig). The highest frustration IC values correspond to canonical contacts where the minimally frustrated state is the conserved one, while those at which the highly frustrated state is the most informative do not display Frustration IC values higher than 0.5 (from a maximum of *log*_2_(3)≃ 1.584). In order to locate the most informative contacts we considered the interactions with Frustration IC values higher than the highest value at the neutral distribution (S2 Table). These interactions mainly connect residues that are more conserved at the sequence level and are important for both the internal stability of the repeats as well for the stabilization of the interactions between neighboring repeats comprising a network of minimally frustrated interactions (Fig 7C). This network is composed of 15 interactions that are internal to each repeat and 8 interactions between adjacent repeats. The GXTPLHLA motif is involved in many of the interactions. Interestingly, some of the conserved interactions are established between residues at the helices and the ones that compose the *β*-hairpins. It is worth noting that a majority of these interactions are formed between hydrophobic residues (S2 Table).

#### Local frustration: The interplay between stability and function

Native states for most proteins are only marginally stable [38]. Energetic conflicts are found in native ensembles and are related to biological functions [15, 39, 40]. We calculated the configurational frustration index over all the protein structures in the dataset. The distributions for the frustration index show differences between repeats located at the termini and the internal ones (S4A Fig), that mainly arise from the solvent exposure of terminal repeats.

We observe an enrichment of highly frustrated clusters when analyzing the whole repeat arrays (S4B Fig) compared to the region including only internal repeats (S4C Fig), indicating that terminal repeats are enriched in highly frustrated interactions. In addition to terminal repeats, we detected an enrichment of highly frustrated interactions at binding sites (S4D Fig) (detected as interacting residues between ANKs and partners at co-crystal structures in the dataset). Also, those residues annotated as insertions within and between repeats are enriched in highly frustrated interactions (S4E Fig). We observe that the amino acids located at the surroundings of regions annotated as deletions are also enriched in highly frustrated interactions (S4F Fig).

### Protein-protein interactions in Ankyrin Repeat Arrays

There are 83 structures from the dataset where the ANKs are in complex, either forming homoligomers or heteroligomers. An entry belongs to the non-redundant set if there is no other complex containing the same ANK Uniprot ID or if it is in complex with a different partner than the entries already included. A total of 33 complexes were analyzed (S3 Table). ANK repeats involve an average of 20% of their residues in binding (sd = 11%). 80% of the binding residues correspond to canonical positions in the repeats (sd = 0.24%), 15% correspond to insertions either between or within repeats (sd = 0.19%) and only 5% can be mapped to non repetitive regions.

**Fig 8:**
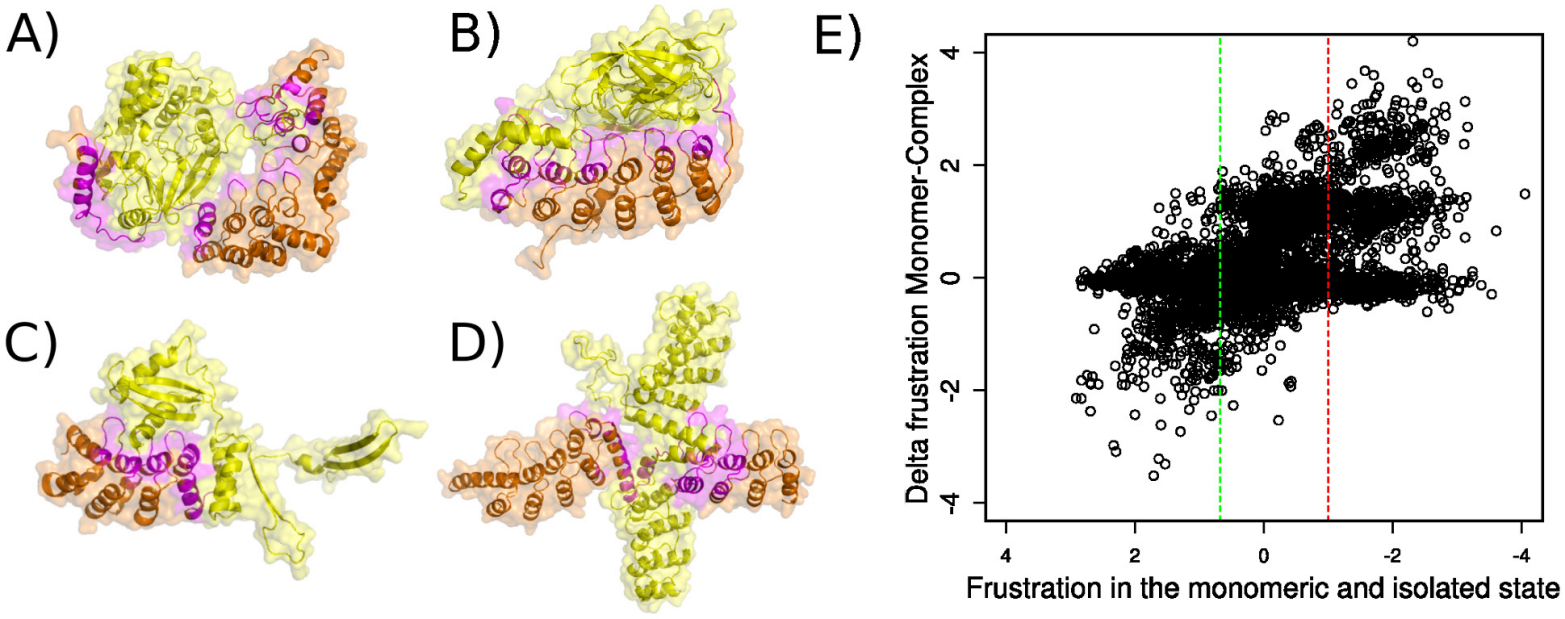
Quaternary assemblies in ANKs. Quaternary assemblies that involve ANKs are shown with the ANK molecule in orange and the rest of the complex in yellow. Residues from the ANK structure that are involved at the interface are coloured in magenta. A) Myosin phosphatase targeting subunit 1 (MYPT1) in complex with the ser/thr phosphatase-1 (delta) (PdbID: 1s70). B) I*κ*B*β*/NF-*κ*B p65 homodimer complex (PdbID: 1k3z) C) YAR1 when binding the 40S ribosomal protein S3 (PdbID: 4bsz) D) Tankyrase-1, (PdbID: 3utm). E) Change in the configurational frustration index between the unbound and bound states for the ANK proteins. In the x-axis the configurational frustration for the monomeric unbound state is shown. In the y-axis the difference between the complexed state and the monomeric state is shown. A positive change means that the frustration is decreased upon interaction.

In general, the whole repeat array is at some point involved in protein-protein interactions (S2B Fig). We observe that interactions are enriched in the *β*-hairpins, as previously described [1]. However the inter helices region is also enriched in interacting residues. Thus there is no conserved mechanism for ANKs binding other proteins (Fig 8A–8D). In addition to a non localized epitope at the canonical structure of repeats, interactions usually involve non repetitive regions, as is the case of the complex between the ANK repeat-containing protein, myosin phosphatase targeting subunit 1 (MYPT1, PdbID: 1s70) and the ser/thr phosphatase-1 (delta) (Fig 8A) where a helix is connected to the repetitive array by a loop which directly interacts with the partner. Another example of this is the I*κ*B*β*/NF-*κ*B p65 homodimer complex (PdbID: 1k3z) (Fig 8B) where a loop with no defined secondary structure interacts with NF-*κ*B. A related protein, I*κ*B*α*, also displays a loop region at the C-terminal that binds the NF-*κ*B P50/P65 heterodimer. This region is disordered in the free protein and has profound implications for the binding energy between the molecules [41]. While in the case of I*κ*B*β* all the repeats are involved in the interaction with the other molecule, in other ANKs like MYPT1 or in the case of the ANK repeat-containing protein YAR1 when binding the 40S ribosomal protein S3 (PdbID: 4bsz) (Fig 8C), the interface is concentrated at specific locations along the array. ANKs are also able to form homodimeric complexes involving different types of interfaces with one of the most interesting cases being the Tankyrase-1 (PdbID: 3utm) (Fig 8D), where the monomers are intertwined in a crossed fashion involving their central repeats, that have extended helices due to insertions. It was previously described that binding sites are enriched in high local frustration [15]. We compared the configurational frustration index calculated in both the unbound ANK monomers and in the bound state (Fig 8E). The frustration distribution for both states are similar, indicating that both correspond to largely minimally frustrated networks of interactions. We compared the frustration change at the contacts established by those residues that are involved at the interface between the ANKs and their partners, and observed that the majority of them, ∼60%, change towards lower frustration values. This can be attributed to the change in the solvent accessibility upon binding that is captured by the burial term in the AMW energy function. The remaining ∼40% of highly varying contacts, change in the opposite direction. We observe that while much of the frustration present at the unbound state is released in the complexed state, some new highly frustrated interactions arise as a product of the interface formation. It is tempting to speculate that the frustration that appears upon oligomerization has functional consequences for further conformational transitions once the complexes are assembled.

## Discussion

In order to characterize the ANK repeat protein family, detection and consistent annotation of repeats within the repetitive arrays is required. The high sequence diversity among repeats and their short lengths pose a problem when trying to accomplish the annotation using sequence-based methods. We applied a method that analyses periodicities and repetitions at protein structures by tiling the structural space [13]. By combining this approach with the relative foldability function [26] we were able to consistently define both the length and the phase of the ANK repeats. The structural detection of ANK repeats allowed us to detect instances that are completely missed when using other structural methods as AnkPred [42] that fails to detect, for example, the sixth repeat on I*κ*B-*α* (PdbID: 1ikn,D) or the repeats within the K1 protein from the Vaccinia virus (PdbID: 3kea,B). While the Console method [43] is able to detect the six repeats on I*κ*B and almost all repeats in the K1 protein, it is not possible to select the phase at which the repeats appear, constituting an obstacle for comparative studies. With our procedure, all repeats from the protein structures in a non-redundant dataset (S1 Table) were consistently annotated, together with the insertions and deletions. The new sequence profiles derived from these annotations, based on a strictly structure-based multiple sequence alignment, give a new perspective about the sequence divergence that ANK repeats can tolerate. Moreover, these profiles could be used in combination with the existent ones in order to improve the annotation and sequence coverage of ANKs at sequence databases such as Pfam, Uniprot and definitively would be useful in order to perform consistent high-quality annotation on structural databases such as RepeatsDB [44] in which all repeat-containing proteins are being deposited and characterized.

We have shown that the N-terminal, internal and C-terminal repeats are different in their sequence and energetic signatures, having distinct secondary structure compositions. Internal repeats display the highest conservation on both sequence and energetic signatures while the N-terminal repeats were the least conserved ones. If sequence conservation is compared to the conservation of local frustration at the structure level, we observe that there exists a positive linear and significant correlation between them for the C-terminal and internal types while no significant correlation was observed for the N-terminal case. This correlation suggests that the more similar to the consensus sequence a repeat is the more foldable it will be. Consensus mutations have proven to be effective at stabilizing proteins [45] with some exceptions [46]. Destabilizing consensus mutations were observed at highly co-varying positions or invariant ones where hidden correlations can occur [4, 47, 48]. We calculated which interactions within the canonical structure of ANK repeat pairs are the most conserved leading to a set of highly conserved residues, at the sequence level, that are connected by a network of minimally frustrated interactions. The majority of these interactions are established between hydrophobic residues. Non hydrophobic interactions involve the GXTPLHLA motif interacting with the *β*-hairpin at the single repeat level or interactions with the loops at adjacent repeats, as well as interactions with their respective GXTPLHLA motifs (S2 Table).

Stabilizing interactions within and in between ANK repeats are encoded in the consensus sequence of a single, self-compatible repeat as evidenced by the success of consensus design by stacking identical repeats [20, 29]. Folding cooperativity of repeat proteins is highly influenced by the intrinsic stabilities of the different repeats and their interfaces. It has been computationally shown that these proteins are ‘poised’ at particular ratios of inter-repeat and intra-repeat interaction energies that allow them to undergo partially unfolding under physiological conditions which would be a requirement to perform their biological functions [49, 50]. We have mapped the interactions that are the most energetically favored between residues composing the canonical structure of ANK repeats. Identifying which of these interactions, within and in between natural repeats, are not satisfied at natural repeat arrays could help trace the determinants of differential folding behaviors between different ANKs which, despite having the same number of repeats, display substantial different dynamical properties.

ANK repeats can undergo several modifications, i.e insertions and deletions at their canonical 33 residues length framework. Analysis of the energetics of the interactions that surround these structural modifications, showed us that there exists an enrichment of highly frustrated interactions around them. This suggests that insertions and deletions occurring at ANK repeats may have functional consequences for the entire molecule. Moreover, binding residues, identified from co-crystallized complexes, showed that these are also enriched in highly frustrated interactions, that are mostly relieved upon binding.

ANKs are adapted to perform their principal function of specifically binding other proteins. Their sequences and structures can vary in order to maximize their recognition properties, introducing considerable structural deviations and even displaying order/disorder transitions in many cases. Their modularity allows for an exquisite tuning of individual repeats giving differential dynamic properties to different regions within the repetitive array. The presence of frustration at binding sites, insertions and deletions shows that, in many cases, evolution seems to have kept energetic conflicts that destabilize the individual ANK structures but at the same time should be critical to drive the recognition process and further release of frustration when complexed. ANKs seem to strategically combine the introduction of structural perturbations by mutation of key residues at the canonical sequence of the different repeats while maintaining some of them unchanged. This tinkering of the sequence not only modifies the global structure and modulate the affinity and specificity for partners recognition, but it may also encode complex dynamic behaviors, as the presence of a multiplicity of folding intermediates or an increased conformational plasticity that arises due to these variations [51].

## Supporting Information

S1 Fig Clustering Anks. Two different metrics were used in order to build a dendogram by clustering the ANKs. A) The relI variable was used as a metric to build the dendogram. ANKs in the same clusters are related by their orthology and paralogy. B) The relS variable was used to construct the dendogram. ANKs in the same cluster have comparable number of repeats- Both dendograms were constructed using the hclust function in the R language. Colours below the dendograms represent clusters detected by the cutreeHybrid function from the dynamicTreeCut R package ([52]).
(TIFF)

S2 Fig Structural properties of canonical ANKs. A) DSSP profiles for the different repeat types: We used the DSSP software in order to measure which were the secondary structure elements at each canonical position for the different repeat types N-terminal repeats (top), Internal repeats (middle), C-terminal repeats (bottom). B) Binding contacts profiles: We calculated the relative abundances of contacts that affect each canonical position at the different repeat types N-terminal repeats (top), Internal repeats (middle), C-terminal repeats (bottom).
(TIFF)

S3 Fig Frustration IC distributions. The Frustration IC is a continuous value, in this figure the distributions are shown. The red curve is the distribution for interactions where the highly frustrated state is the most informative, the gray distribution is the one for interactions where the neutral state is the most informative one and the green distribution accounts for values where the minimally frustrated state is the most informative one.
(TIFF)

S4 Fig Distribution of frustration in the ankyrin repeat. (A) Configurational frustration index calculated separately for N-terminal (orange), internal (red) and C-terminal repeats (cyan), we observe how the internal repeats differ from the terminal ones. (B) Pair distribution function calculated for those residues comprised within the first and last repeats detected over the structures. (C) Pair distribution function calculated over internal repeats, only. (D) Pair distribution function calculated over residues that are in contact with protein partners at known crystal complexes. (E) Pair distribution function calculated over residues that belong to insertions within the ankyrin repeats. (F) Pair distribution function calculated over the residues that are immediately before and after the detected deletions within repeats.
(TIFF)

S1 Table Non redundant dataset of Ankyrin Repeat Protein Structures. (TEX)

S2 Table Minimally frustrated interactions network in canonical ANKs. (TEX)

S3 Table Non redundant dataset of ANK complexes structures. (TEX)
