# Supplementary figures and images for: Structural and Energetic Characterization of the Ankyrin Repeat Protein Family
Source: PLoS Comput Biol. 2015 Dec 21;11(12):e1004659. doi: 10.1371/journal.pcbi.1004659 (PMC4687027; doi:10.1371/journal.pcbi.1004659)

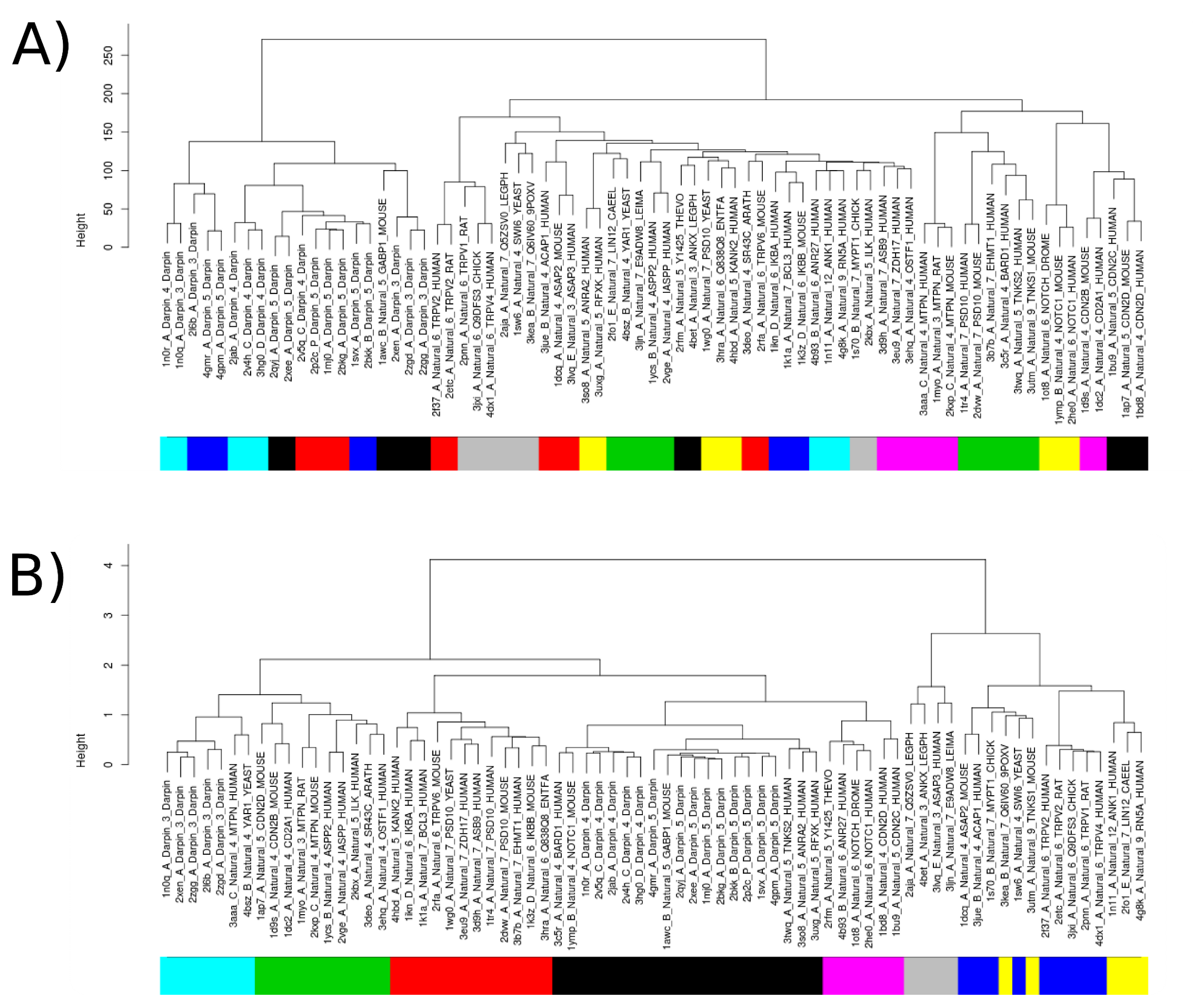

Supplement: S1 Fig — Two different metrics were used in order to build a dendogram by clustering the ANKs. A) The relI variable was used as a metric to build the dendogram. ANKs in the same clusters are related by their orthology and paralogy. B) The relS variable was used to construct the dendogram. ANKs in the same cluster have comparable number of repeats- Both dendograms were constructed using the hclust function in the R language. Colours below the dendograms represent clusters detected by the cutreeHybrid function from the dynamicTreeCut R package ([52]). (TIFF) [file pcbi.1004659.s001.tiff]

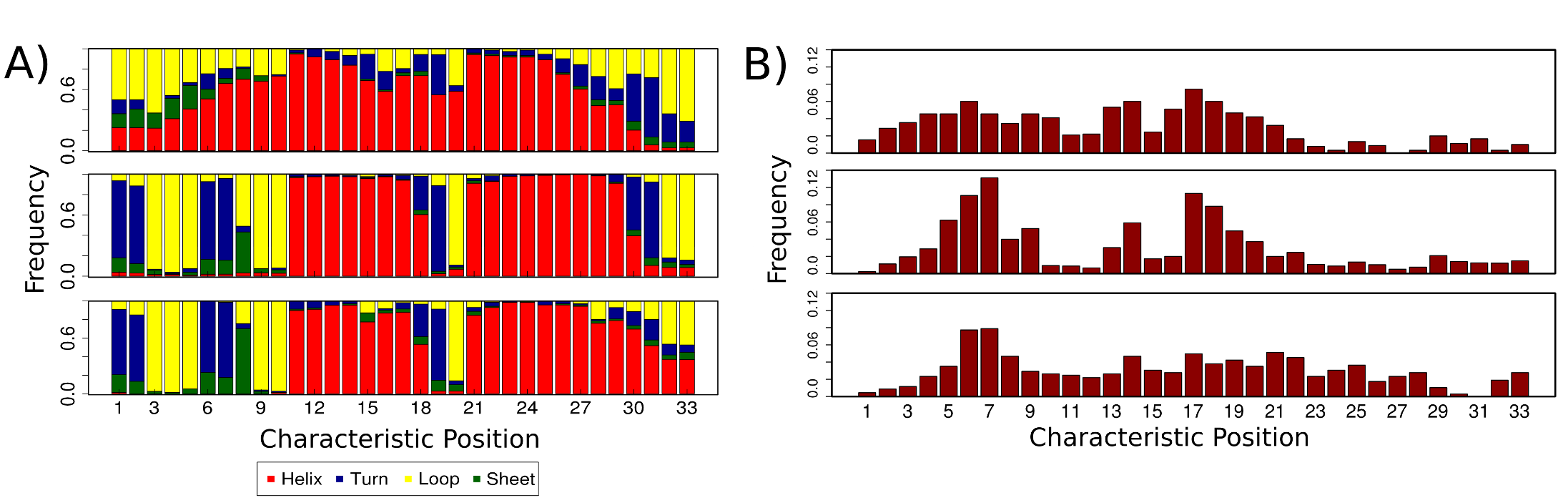

Supplement: S2 Fig — A) DSSP profiles for the different repeat types: We used the DSSP software in order to measure which were the secondary structure elements at each canonical position for the different repeat types N-terminal repeats (top), Internal repeats (middle), C-terminal repeats (bottom). B) Binding contacts profiles: We calculated the relative abundances of contacts that affect each canonical position at the different repeat types N-terminal repeats (top), Internal repeats (middle), C-terminal repeats (bottom). (TIFF) [file pcbi.1004659.s002.tiff]

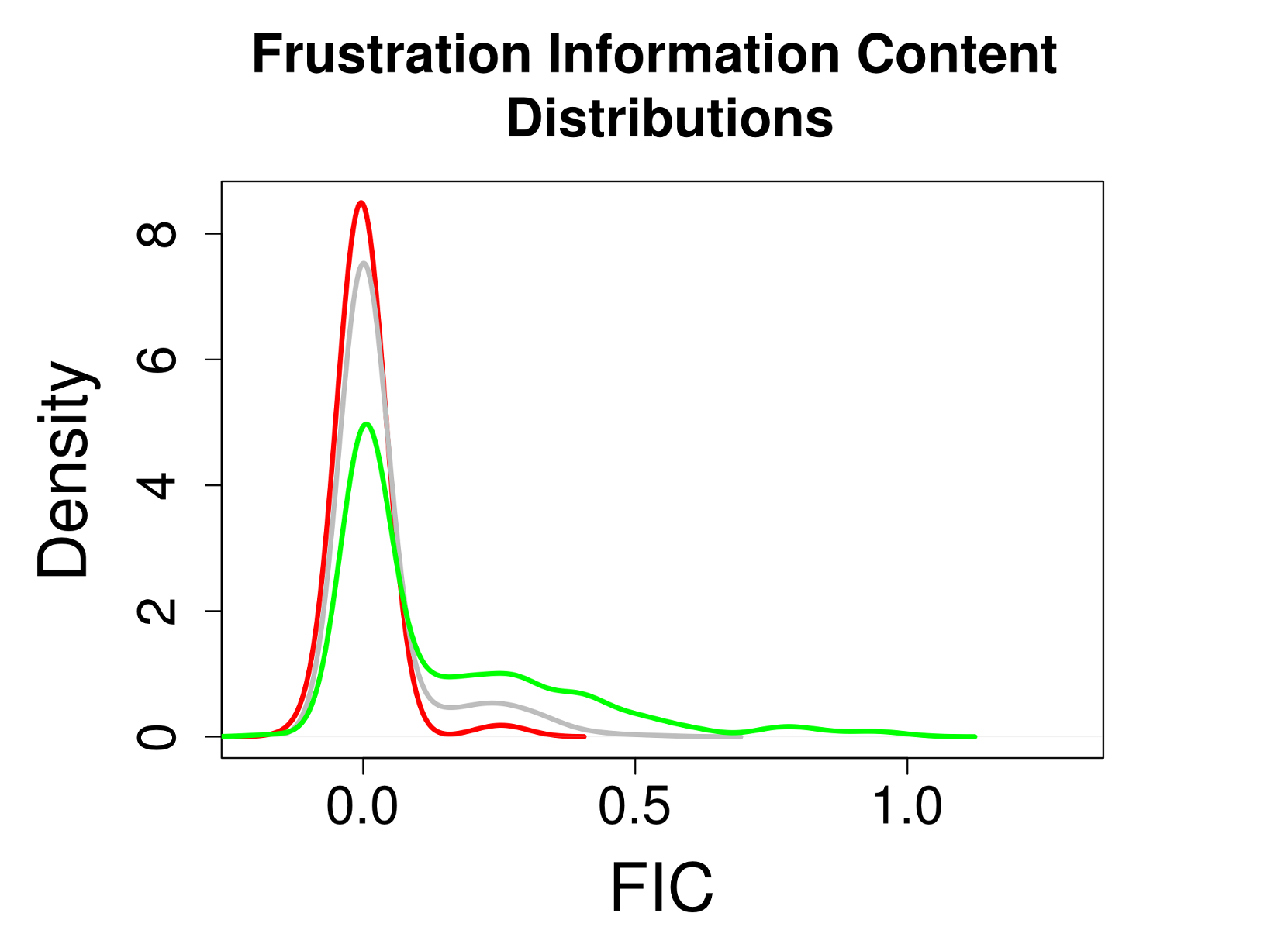

Supplement: S3 Fig — The Frustration IC is a continuous value, in this figure the distributions are shown. The red curve is the distribution for interactions where the highly frustrated state is the most informative, the gray distribution is the one for interactions where the neutral state is the most informative one and the green distribution accounts for values where the minimally frustrated state is the most informative one. (TIFF) [file pcbi.1004659.s003.tiff]

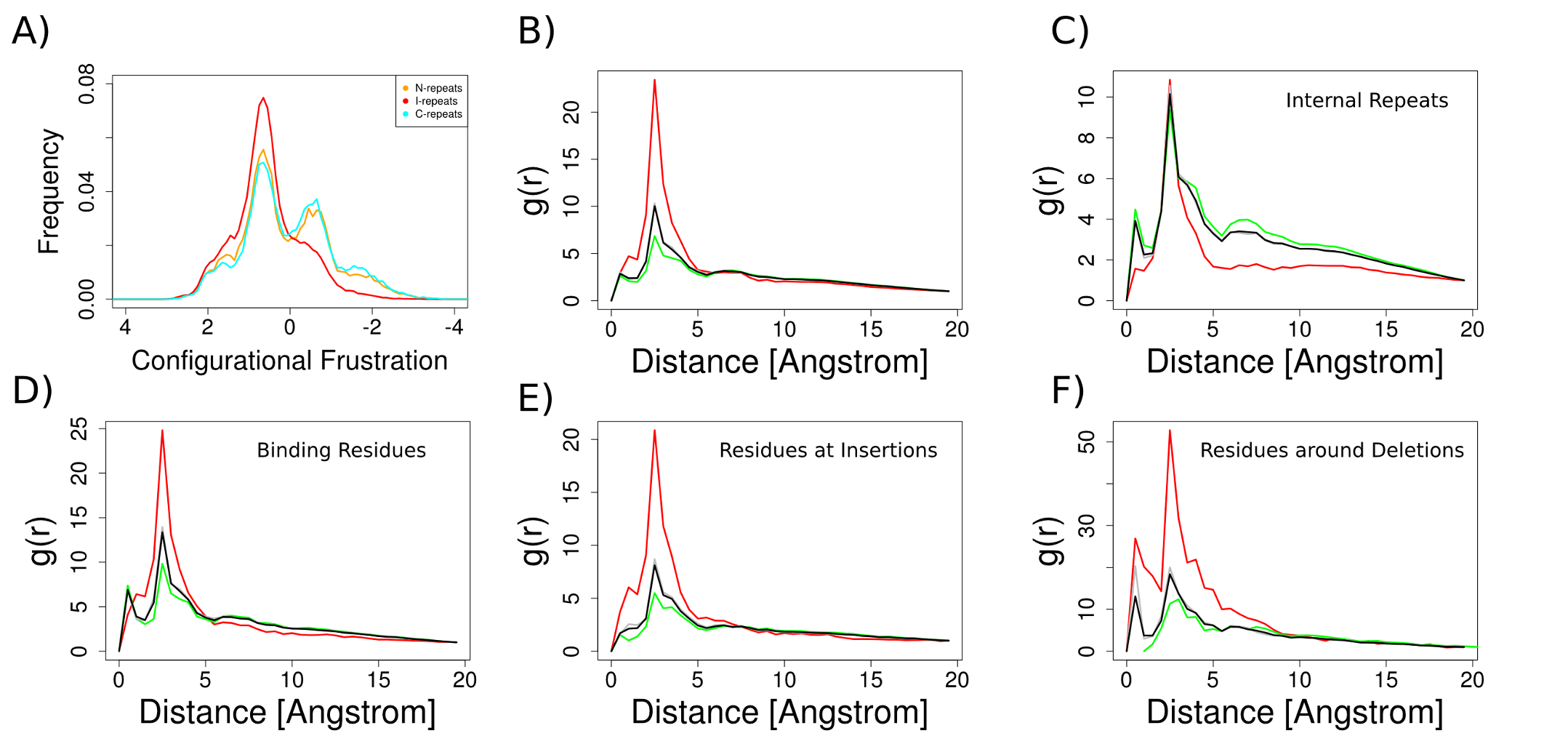

Supplement: S4 Fig — (A) Configurational frustration index calculated separately for N-terminal (orange), internal (red) and C-terminal repeats (cyan), we observe how the internal repeats differ from the terminal ones. (B) Pair distribution function calculated for those residues comprised within the first and last repeats detected over the structures. (C) Pair distribution function calculated over internal repeats, only. (D) Pair distribution function calculated over residues that are in contact with protein partners at known crystal complexes. (E) Pair distribution function calculated over residues that belong to insertions within the ankyrin repeats. (F) Pair distribution function calculated over the residues that are immediately before and after the detected deletions within repeats. (TIFF) [file pcbi.1004659.s004.tiff]
